# The N-terminal domain of *Mycobacterium tuberculosis* PPE17 (Rv1168c) protein plays a dominant role in inducing antibody responses in active TB patients

**DOI:** 10.1371/journal.pone.0179965

**Published:** 2017-06-26

**Authors:** Philip Raj Abraham, Niteen Pathak, Gourango Pradhan, Gaddam Sumanlatha, Sangita Mukhopadhyay

**Affiliations:** 1 Laboratory of Molecular Cell Biology, Centre for DNA Fingerprinting and Diagnostics (CDFD), Hyderabad, India; 2 Graduate Studies, Manipal University, Manipal, India; 3 Bhagwan Mahavir Medical Research Center, Hyderabad, India; University of Hyderabad, INDIA

## Abstract

The PPE (proline-proline-glutamic acid) proteins of *Mycobacterium tuberculosis* are characterized by a conserved N-terminal domain of approximately 180 amino acids and variable C-terminal domain. Since last decade, these proteins have gained much importance in the serodiagnosis of tuberculosis (TB) as they act as a source of antigenic variation. We have demonstrated earlier that one of the PPE proteins PPE17 (Rv1168c) induces strong B-cell and T-cell responses in active TB disease and also displays a higher antibody titer compared to immunodominant antigens such as ESAT-6, Hsp60 and PPD. However, the immunodominant domain of PPE17 (N-terminal or C-terminal) was not examined in detail. In the present study, we observed that antibody responses elicited in TB patients were directed mostly towards the N-terminal domain of PPE17 (N-PPE17). The antibody generated against N-PPE17 in TB patients did not significantly cross-react with N-terminal domains of other PPE proteins used in this study. Our data suggest that the N-terminal domain of PPE17 protein is immunodominant and could be used as a better serodiagnostic marker than the full-length PPE17 protein.

## Introduction

Despite the fact that the disease tuberculosis (TB) can be cured, it remains one of the world’s biggest threats accounting for millions of deaths every year. The World Health Organization (WHO) has estimated 10.4 million new cases of active TB and 1.8 million deaths due to TB in 2015 [1]. The current situation has become more complicated due to emergence of multi and extensively drug resistant strains of *M*. *tuberculosis* and appearance of co-infection of human immunodeficiency virus (HIV) with TB. TB together with HIV infection ranks as a leading cause of death worldwide. Currently available methods for diagnosis of active TB have several limitations. For example, sputum smear microscopy requires highly trained manpower and diligence but sensitivity of detection is not satisfactory in patients co-infected with HIV [2]. *In vitro* mycobacterial culture, the gold standard for TB diagnosis, not only requires complex media formulations but also cumbersome, technically demanding and more importantly time consuming [3,4]. Purified protein derivative (PPD) based tuberculin skin test often fails to differentiate BCG (Bacille Calmette-Guérin) vaccinated healthy individuals from active TB patients and it cross-reacts with other mycobacterial species [5]. Over the years, significant efforts have been made to develop rapid TB diagnosis tests. Among these, polymerase chain reaction (PCR) for amplification of *M*. *tuberculosis* specific genes has attracted considerable interest, due its ability to identify *M*. *tuberculosis* in respiratory and non-respiratory specimens in relatively shorter time. However, the usefulness of this method is limited by the presence of PCR inhibitors in clinical specimens and the type of tissue from which DNA is isolated [6,7]. Recently, U.S. Food and Drug Administration approved interferon gamma (IFN-γ) release assay (IGRA) that can aid in the diagnosis of *M*. *tuberculosis* infection. In spite of advantages over the available diagnostic methods, this method still suffers from lack of consistency and reproducibility [8]. Of late, WHO recommended GeneXpert MTB/RIF test that can simultaneously detect TB and rifampicin drug resistance in less than two hours, but its higher operative cost and need of expertise for data analysis make it difficult to be popularized in developing and underdeveloped countries [9].

Measuring the antibody response to *M*. *tuberculosis* antigens by serological assays are appears to be more effective as these tests are simple, robust, cost-effective, shorter turn-around time and require minimal manpower training [10,11]. In addition, these tests can be developed as point-of-care tests that can be implemented at primary health centers in resource-limited countries. Serological assays are considered to be useful in detection of cases like sputum smear-negative TB, extrapulmonary TB, childhood TB and latent TB which are difficult to diagnose by conventional methods [3–8]. Therefore, studies are initiated to identify *M*. *tuberculosis* proteins that can provide enough sensitivity and specificity to be used as a first-line screening tool for serodiagnosis of TB. Various studies indicate that PPE proteins induce B-cell responses in active TB patients and can be used for serodiagnosis of TB [12–22]. One of the PPE proteins, PPE17 (Rv1168c) is shown to be up-regulated in conditions that mimic the macrophage environment features [23–26]. Also, over expression of this protein is observed in macrophages infected with various clinical isolates of *M*. *tuberculosis* [27]. Interestingly, PPE17 is reported to be surface exposed and immunogenic [28] and genes that are homologous to PPE17 are found to be absent in the non-tuberculous mycobacterial species. We reported earlier that PPE17 could discriminate patients with active TB from BCG-vaccinated healthy individuals [15,22]. Interestingly, we observed that PPE17 displayed higher sensitivity in detecting extrapulmonary and smear negative pulmonary TB cases than early secreted antigenic target 6 protein (ESAT-6) and purified protein derivative (PPD) as well as other PPE proteins [14–18,21,22]. We also demonstrated that PPE17 is a potent T-cell antigen which elicited stronger gamma interferon response as compared to PPD [15]. These features make PPE17 a potential candidate antigen for accurate diagnosis of all the clinically distinct categories of active TB.

However, it is unknown which region of PPE17 is immunogenic and can be used as a marker for serodiagnosis of TB. Further, it is well known that the PPE family proteins of *M*. *tuberculosis* shares a conserved N-terminal region of approximately 180 amino acids and variable C-terminal domain [29]. Thus, it is interesting to study if antibodies specific to the N-terminal domain of PPE17 (N-PPE17) show serological cross-reactivity with N-terminal domains of other PPE proteins in TB patients. Therefore, in the present study, we measured the levels of antibodies against the N-terminal domain of PPE17 in sera collected from active TB patients. The findings presented herein can be helpful to further design peptide based test for serodiagnosis of active TB cases.

## Materials and methods

### Ethics statement

The study was approved by the Institutional Ethical Committee of the Mahavir Hospital and Research Centre (No. ECR/450/inst/AP/2013). Written informed consent was obtained from the study participants before collection of sera samples.

### Sera collection

Sera were collected from patients diagnosed for active tuberculosis at the DOTS (directly observed treatment short course) centre of Mahavir Hospital and Research Centre, Hyderabad, India. A total of 180 samples were collected from active TB patients showing symptoms for pulmonary and extrapulmonary TB (Table 1).

**Table 1:** Characteristics of study participants.

Characteristics	No. of Participants
**Gender**	
Male	76
Female	104
**Age (years)**	
Below 14	9
15–24	63
25–34	51
35–44	37
45–54	12
55–64	6
65 and above	2
**Clinical category**	
Pulmonary TB	125
Smear positive	78
Smear negative	47
Extrapulmonary TB	55
**Total**	180

These patients were diagnosed based on the guidelines of the Revised National TB Control Programme of India. Parameters used for the diagnosis of pulmonary TB were smear microscopy, chest radiography and observation of clinical symptoms suggestive of TB while the extrapulmonary cases were confirmed by chest radiography, tissue biopsy and clinical symptoms (http://www.tbcindia.org). Sera samples were also collected from BCG-vaccinated healthy individuals (n = 20). In this control group, 14 were male and 6 were female participants between 24 to 45 years of age. No symptoms suggestive of clinically active TB were present in these BCG-vaccinated healthy individuals during collection of serum samples. Both the categories of study participants tested negative for human immunodeficiency virus infection.

### Purification of recombinant PPE proteins

The full-length PPE17 protein of *M*. *tuberculosis* is 346 amino acids in length and is characterized by the presence of a conserved N-terminal domain of approximately 180 amino acids (S1 Fig). The expression and purification of recombinant full-length PPE17 protein (PPE17) was carried out following the methods as described by us earlier [15,22]. For purification of N-terminal domain of PPE17 (N-PPE17) protein, ORFs corresponding to 1 to 173 amino acids was amplified from BAC library contig Rv71 (C2) [kindly provided by Dr. Shekhar C. Mande, CDFD, Hyderabad] using forward primer 5' ATGGATCCATGGATTTCACAATTTTTCCGCCGG 3' (*BamH1*) and reverse primer 5' ATAAGCTTCTACGGGTTGGCGATCGGCGC 3' (*HindIII)*. The amplified products were cloned in frame with N-terminal poly-histidine tag in pRSET-A vector (Invitrogen, Carlsbad, CA, USA). After confirming the clones by PCR and sequencing, the clones were transformed in *Escherichia coli* BL21 (DE3) pLysS cells. The full-length PPE18 (Rv1196) was cloned in frame with N-terminal poly-histidine tag in pRSET-A vector (Invitrogen, Carlsbad, CA, USA) and purified as described by us earlier [30]. Genes encoding full-length PPE44 (Rv2770c) and full-length PPE65 (Rv3621c) genes were cloned in frame with C-terminal poly-histidine tag in pET-23a vector and transformed in *E*. *coli* BL21 (DE3) pLysS cells. All these proteins were purified under native condition as described by us earlier [15, 22, 30]. Briefly, primary cultures were grown in Terrific broth (Becton Dickinson, Difco, Sparks, MD) containing ampicillin (100 μg/ml) at 37°C overnight. The secondary cultures were grown until the optical density (OD) of the culture reaches to 0.4 to 0.5 at 600 nm, induced with 1 mM isopropyl-β-D-thiogalactopyranoside (Sigma Aldrich, MO, USA), and further incubated at 37°C for 3 hr. The culture pellets were resuspended in lysis buffer (PBS containing 1 mg/ml lysozyme and 1% sodium lauryl sarcosine), sonicated, and centrifuged. Supernatant was loaded on TALON resin column (Takara Bio Inc. CA, USA) and kept for binding at 4°C for 30 min. The column was washed with PBS containing 20 mM imidazole. Protein fractions were eluted in elution buffer (200 mM imidazole) and the purity of the recombinant proteins were confirmed by Coomassie blue staining (S2 Fig).

The purified proteins were dialyzed in phosphate buffered saline (PBS, pH 7.4) and concentration of the proteins was estimated using bicinchoninic acid kit (Micro BCA protein assay kit from Thermo Scientific, Rockford, IL, USA). The molecular weight of PPE17, N-PPE17, PPE18, PPE44 and PPE65 was about 40 kDa, 27 kDa 45 kDa, 42 kDa and 43 kDa respectively.

### Enzyme Immuno Assay (EIA)

The serum IgG levels in various sera samples were quantified following EIA, as described earlier [15,22]. Briefly, each well of the 96-well microtiter plate (Costar, Corning, NY, USA) was coated with 50 μl of PBS containing 1 μg of PPE17 (500 nM) or N-PPE17 (740 nM) recombinant protein and the plates were incubated overnight at 4°C. Next day, after washing with PBS, the wells were blocked with 200 μl of blocking buffer (PBS containing 2% bovine serum albumin) and kept at 37°C for 2 hr. After washing the EIA plates for three times with PBS-Tween buffer (PBS-T), sera samples (50 μl) were added and the plates were incubated for 1 hr at 37°C. Subsequently, 50 μl of anti-human immunoglobulin G (IgG) conjugated to horseradish peroxidase (HRP, Sigma Aldrich, MO, USA) was added at 1/8000 dilution and the plates were incubated for 1 hr at 37°C. After washing the wells, the HRP activity was measured by *o*-phenylenediamine tetrahydrochloride using H_2_O_2_ as substrate. The reaction was terminated with 1N H_2_SO_4_ and absorbance values were measured at 492 nm with an EIA reader (BioTek Instruments Inc., VT, USA). For inhibition EIA, the sera were pre-incubated with 1 μg/50 μl of recombinantly purified full-length PPE17 (500 nM) or PPE18 (444 nM) or PPE44 (476 nM) or PPE65 (465 nM) protein at 37°C for 1 hr in 96 well flat bottom micro test plates (Tarsons Products Pvt. Ltd, India) and then added directly to the N-PPE17 pre-coated plates and EIA was carried out.

### Statistical analysis

The mean EIA absorbance values (OD at 492 nm (A_492_)) of the healthy control sera plus three standard deviations was calculated and used as cut-off values. A sample showing antibody levels greater than or equal to the cut-off value was considered as positive. For calculating the statistical significance between two groups, non-parametric Mann-Whitney test was performed. The analysis of variance (ANOVA) was performed for calculating statistical significance between different clinical categories of TB patients (smear positive, smear negative and extrapulmonary TB) as described previously [15, 22]. Statistical analyses were performed using GraphPad prism and *P* < 0.05 was considered as significant.

## Results

### Both full-length PPE17 (PPE17) and N-terminal domain of PPE17 (N-PPE17) proteins had higher antibody titers in the sera obtained from active TB patients

**Fig 1:**
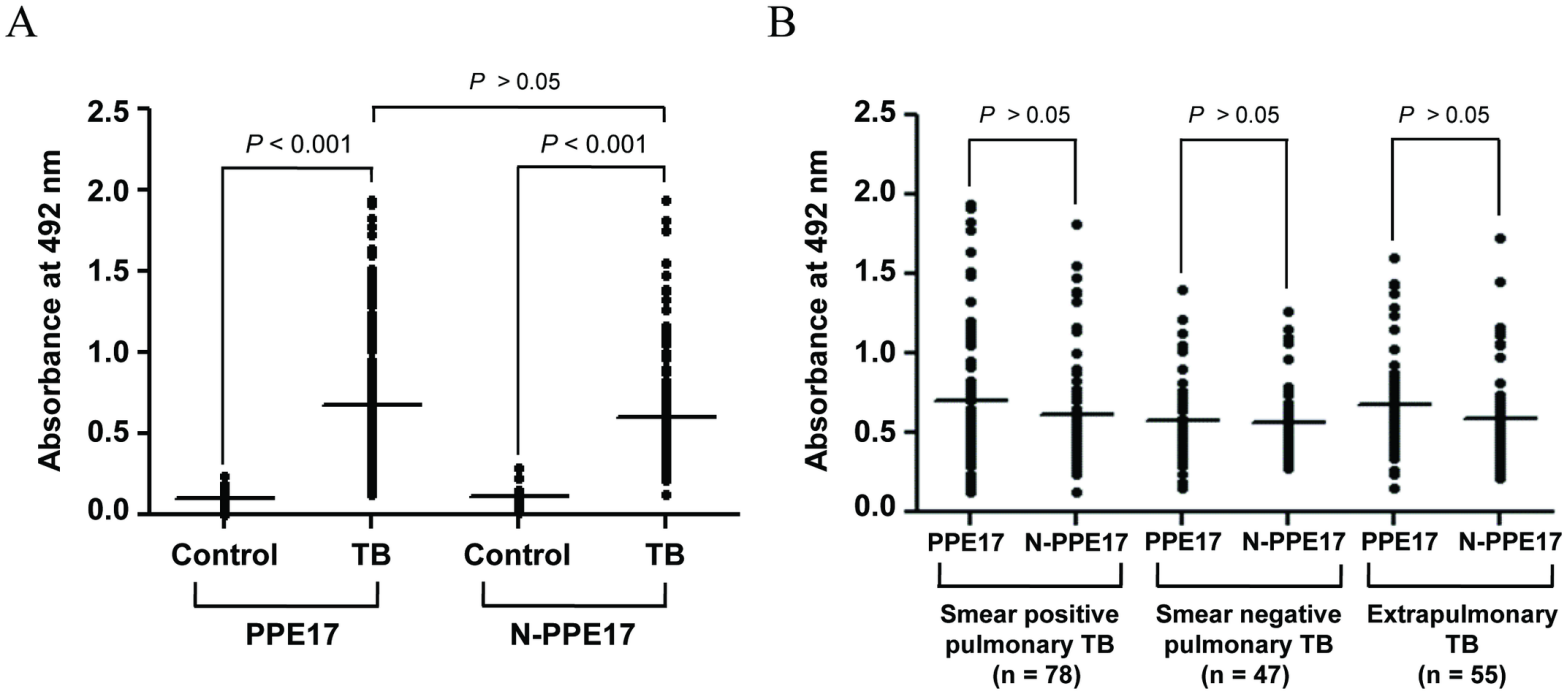
The full-length PPE17 protein (PPE17) as well as the N-terminal fragment of PPE17 (N-PPE17) can discriminate active TB patients (n = 180) from BCG-vaccinated healthy controls (n = 20). (A) The mean absorbance values (OD_492_) of TB patients *versus* healthy controls were compared for PPE17 and N-PPE17 proteins following EIA. (B) The EIA absorbance values shown in Fig 1A were re-plotted to compare the antibody levels of the smear positive pulmonary TB (n = 78), smear negative pulmonary TB (n = 47) and extrapulmonary TB (n = 55) patients between the PPE17 and N-PPE17 proteins. Horizontal line indicates the mean absorbance values.

**Fig 2:**
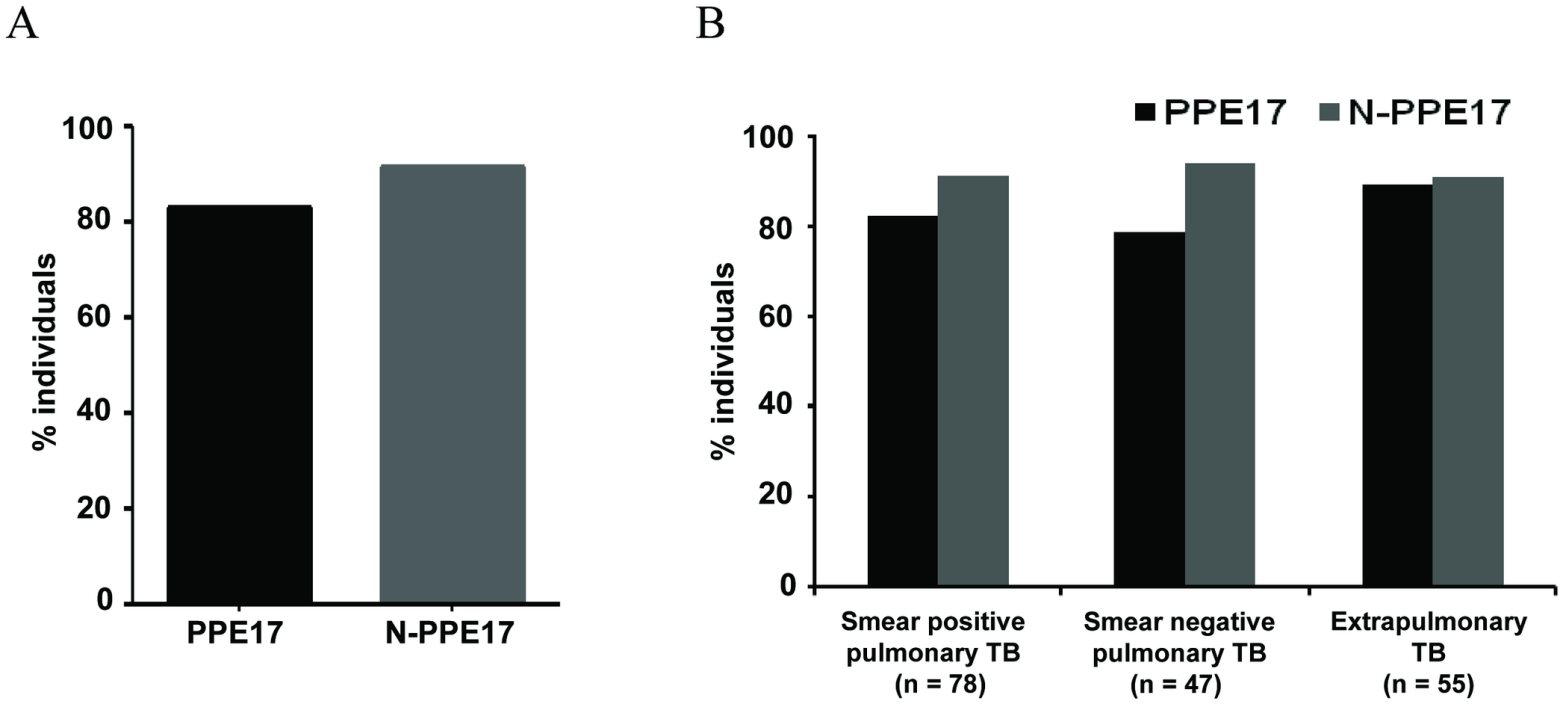
The N-terminal fragment of PPE17 (N-PPE17) is more sensitive to detect TB cases than full-length PPE17 protein. (A) The percentages of high-level responders (patients showing antibody levels greater than or equal to the cut-off values) shown in Fig 1A were calculated using a cut-off value (mean OD_492_ of healthy control sera plus 3 SD) for PPE17 and N-PPE17. (B) The percentage of high level responders was compared for pulmonary (smear-positive and smear-negative) and extrapulmonary TB cases.

When sera obtained from TB patients were incubated with either PPE17 or N-PPE17 protein and compared with that of BCG-vaccinated healthy controls, significantly higher antibody titers were observed in TB patients as compared to healthy controls (*P* < 0.001; Fig 1A). It is interesting to note that the sera from TB patients had PPE17-specific antibody levels similar to that of N-PPE17 (mean OD_492_ ± SEM, 0.676 ± 0.028 for PPE17 and 0.601 ± 0.022 for N-PPE17 respectively). We have also compared the antibody responses against PPE17 and N-PPE17 in different clinical categories such as smear positive, smear negative and extrapulmonary TB. Interestingly, PPE17 as well as N-PPE17 exhibited almost similar antibody titers in the sera obtained from different categories of TB patients (Fig 1B). We did not observe any significant differences (*P* > 0.05) in the antibody responses among these categories of TB cases when full-length PPE17 and N-PPE17 was used in EIA (Fig 1B) indicating that seropositivity did not differ among subtypes of TB patients. To test the sensitivity of PPE17 *versus* N-PPE17, a cut-off value was determined by calculating mean OD_492_ of the healthy control sera plus three standard deviations. Accordingly, the cut-off values were found to be 0.327 for PPE17 and 0.309 for N-PPE17. N-PPE17 displayed marginally higher sensitivity (91.66%; 165/180) compared to PPE17 (83.33%; 150/180) for the diagnosis of TB cases (Fig 2A) and 30 (16.66%) and 15 (8.33%) patients displayed OD below the cut-off value for PPE17 and N-PPE17 respectively.

We have also tested the sensitivities of these antigens for the diagnosis of different clinical categories of TB patients like smear positive TB, smear negative TB and extrapulmonary TB. Interestingly, we observed that the diagnostic sensitivity of N-PPE17 for smear negative and smear positive TB was higher than PPE17 (91%, 93.62% and 90.91% *versus* 80.77%, 78.72% and 89% for smear positive, smear negative and extrapulmonary TB respectively) (Fig 2B).

### Sera from patients with active tuberculosis had antibodies predominantly against the N-terminal fragment of PPE17

**Fig 3:**
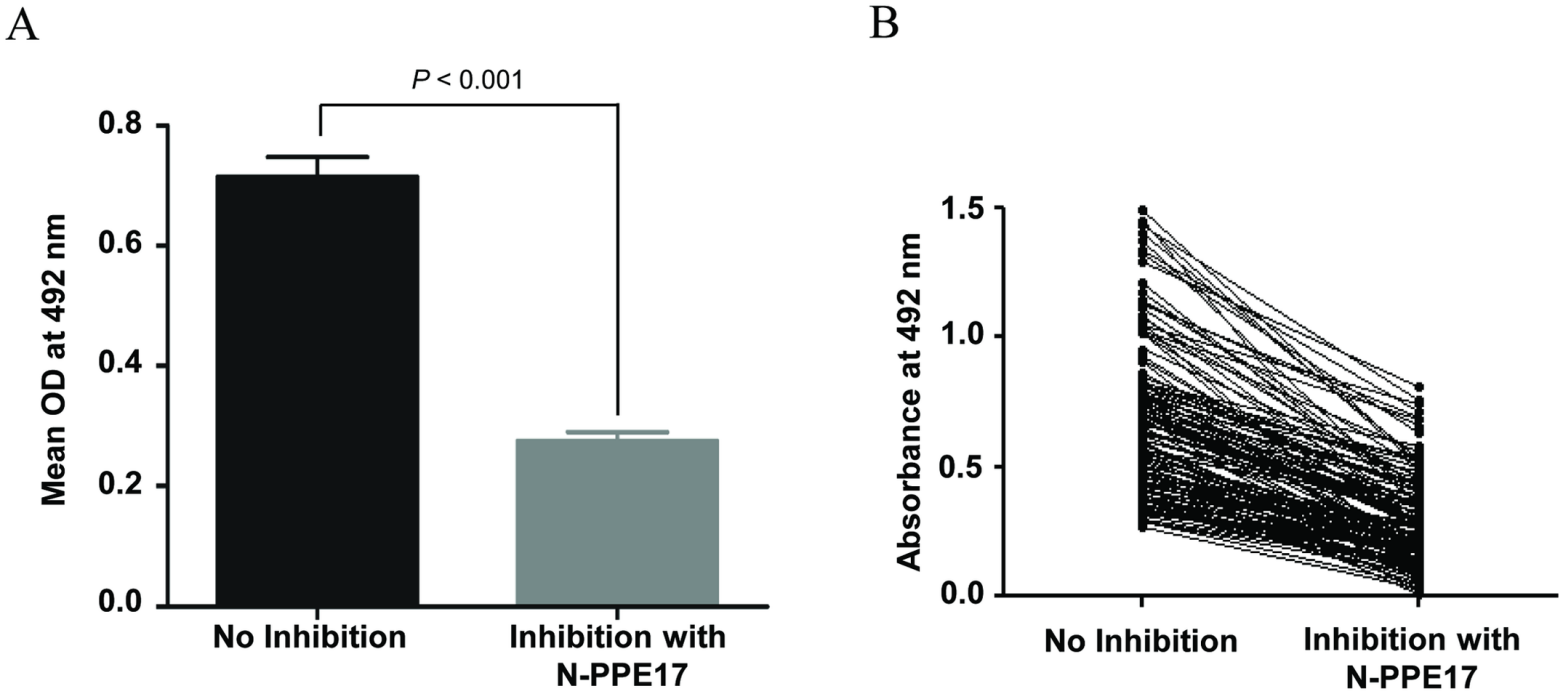
The N-terminal fragment of PPE17 (N-PPE17) is predominantly recognized by the sera of TB patients. (A) The EIA plates coated with full-length PPE17 protein were incubated with TB patients sera (n = 143) that were pre-incubated with 1 μg of N-PPE17 protein diluted in PBS followed by incubation with anti-human IgG-HRP. The absorbance was read at 492 nm using the chromogenic substrate OPD. (B) The EIA absorbance values at 492 nm shown in Fig 3A were re-plotted for individual sera samples of active TB patients.

Next, we tested whether antibody responses against the full-length PPE17 were induced predominantly against the N-terminal domain of PPE17 protein. To test this, we followed inhibition EIA where sera (50 μl) of TB patients were pre-incubated with 1 μg of N-PPE17 protein before the sera were added to the EIA plate coated with PPE17 protein. Interestingly, we observed that pre-incubation of sera samples with N-PPE17 protein significantly inhibited the antibody responses to PPE17 protein (mean OD_492_ ± SEM, 0.714 ± 0.032 *versus* 0.275 ± 0.014; *P* < 0.001; Fig 3A). When Fig 3A was reanalyzed to examine reactivity of individual sample, it was observed that responses of majority of sera samples to PPE17 full-length protein were inhibited by its N-terminal fragment (Fig 3B). The N-PPE17 inhibited antibody responses to PPE17 at both 1 μg/50 μl and 3 μg/50 μl concentrations (S3 Fig).

**Fig 4:**
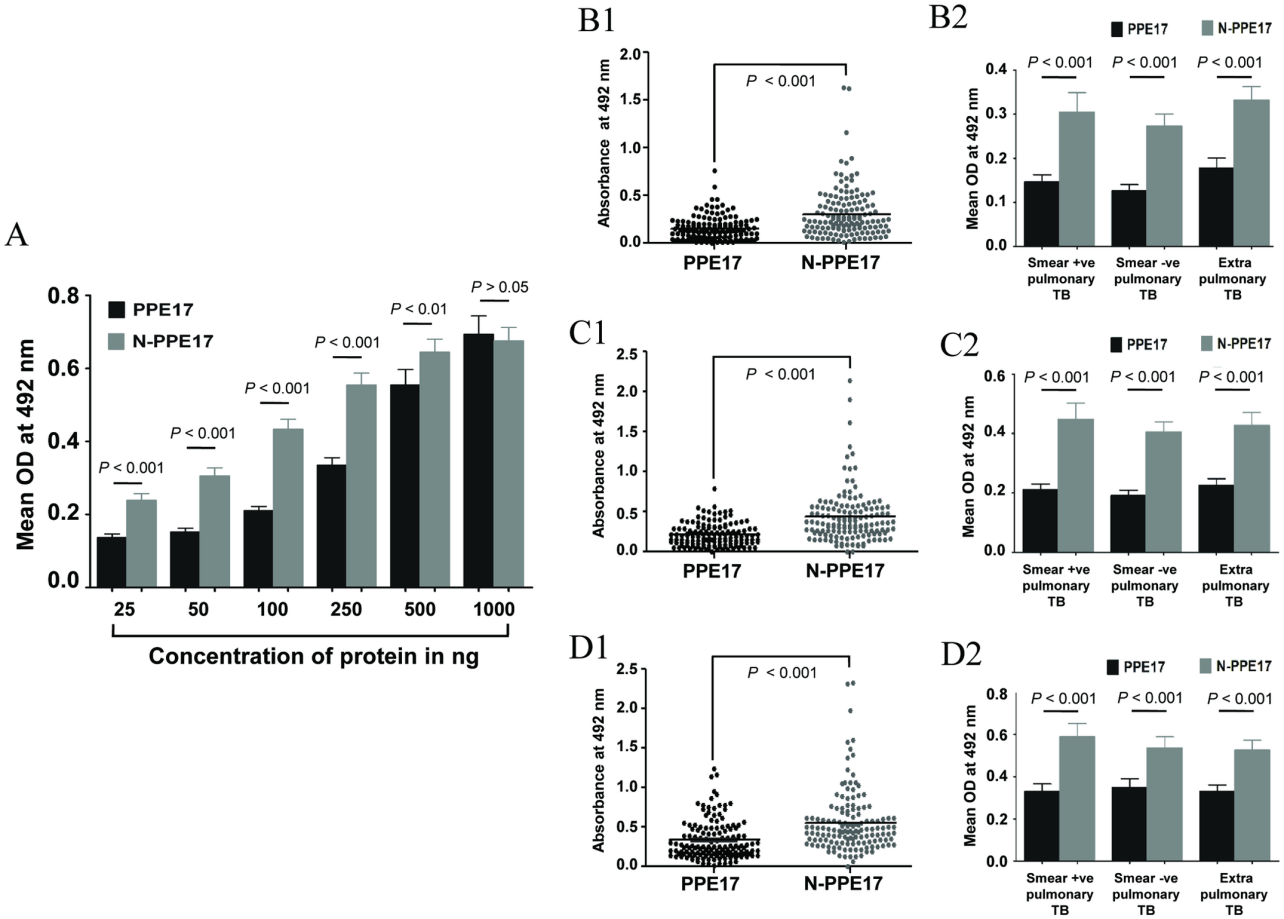
The N-terminal fragment of PPE17 (N-PPE17) shows better seroreactivity than full-length PPE17 in active TB patients (n = 143). (A) The full-length PPE17 or N-PPE17 protein was coated on EIA plate at various concentrations starting from 25 ng/well/50 μl to 1 μg/well/50 μl and antibody responses of TB patient sera were compared. The negative control (no antigen) displayed a background absorbance value of 0.056. Data shown in Fig 4A were re-plotted to compare the antibody levels of TB patients between PPE17 and N-PPE17 at 50 ng/well/50 μl (B1), 100 ng/well/50 μl (C1) and 250 ng/well/50 μl (D1) antigen coating concentrations. Data from Fig 4A were re-plotted to compare the PPE17 and N-PPE17-specific antibody levels in sera samples of smear positive (n = 58), smear negative (n = 38) and extrapulmonary TB (n = 47) patients at antigen concentration of 50 ng/well/50 μl (B2), 100 ng/well/50 μl (C2) and 250 ng/well/50 μl (D2).

To compare the efficacy of PPE17 and N-PPE17 in detecting the TB patient sera, EIA plates were coated with different concentrations (25 ng to 1 μg per well) of either PPE17 or N-PPE17 and then incubated with sera collected from active TB patients and the absorbance values were positively correlated with increased concentrations of PPE17 as well as N-PPE17. Both PPE17 and N-PPE17 proteins displayed higher mean absorbance values at 1 μg/well/50μl concentration. However, N-PPE17 displayed higher mean absorbance values compared to PPE17 at 50 ng/well/50 μl, 100 ng/well/50 μl, 250 ng/well/50 μl and 500 ng/well/50 μl concentrations (Fig 4A). Since, the molecular weight of N-PPE17 is smaller than PPE17, it may be argued that, higher OD observed for N-PPE17 may be due to higher number of molecules present for a given mass of both the proteins. However, we could observe that the mean OD of N-PPE17 at 37 nM (50 ng/well) is higher than the full-length PPE17 at 50 nM (100 ng/well). Similarly, at molar concentrations of 74 nM (100 ng/well) and 185 nM (250 ng/well), N-PPE17 displayed higher OD than the full-length PPE17 protein used at 125 nM (250 ng/ml) and 250 nM (500 ng/ml) respectively. Therefore, N-PPE17 appears to be more efficient to detect antibody levels in the patient sera than the full-length PPE17 protein.

When we compared N-PPE17 and PPE17-specific antibody levels in each sample using antigen concentrations 50 ng/well/50 μl, 100 ng/well/50 μl and 250 ng/well/50 μl, majority of the samples displayed higher absorbance values against N-PPE17 than PPE17 at all the three concentrations used (Fig 4B1–4D1). We also compared the antibody levels in the sera of smear positive, smear negative and extrapulmonary TB cases against PPE17 and N-PPE17 at these concentrations. It was found that though all the categories of patient sera had significantly higher antibody titers against N-PPE17 than full-length PPE17 (*P* < 0.001), no significant differences in antibody responses were observed between these subsets of cases at any antigen concentration tested (*P* > 0.05, Fig 4B2–4D2) indicating that PPE17 as well as N-PPE17 do not discriminate between smear positive, smear negative and extrapulmonary TB cases serologically.

### N-PPE17 did not share cross-reactive epitopes with N-terminal domains of other PPE proteins

**Fig 5:**
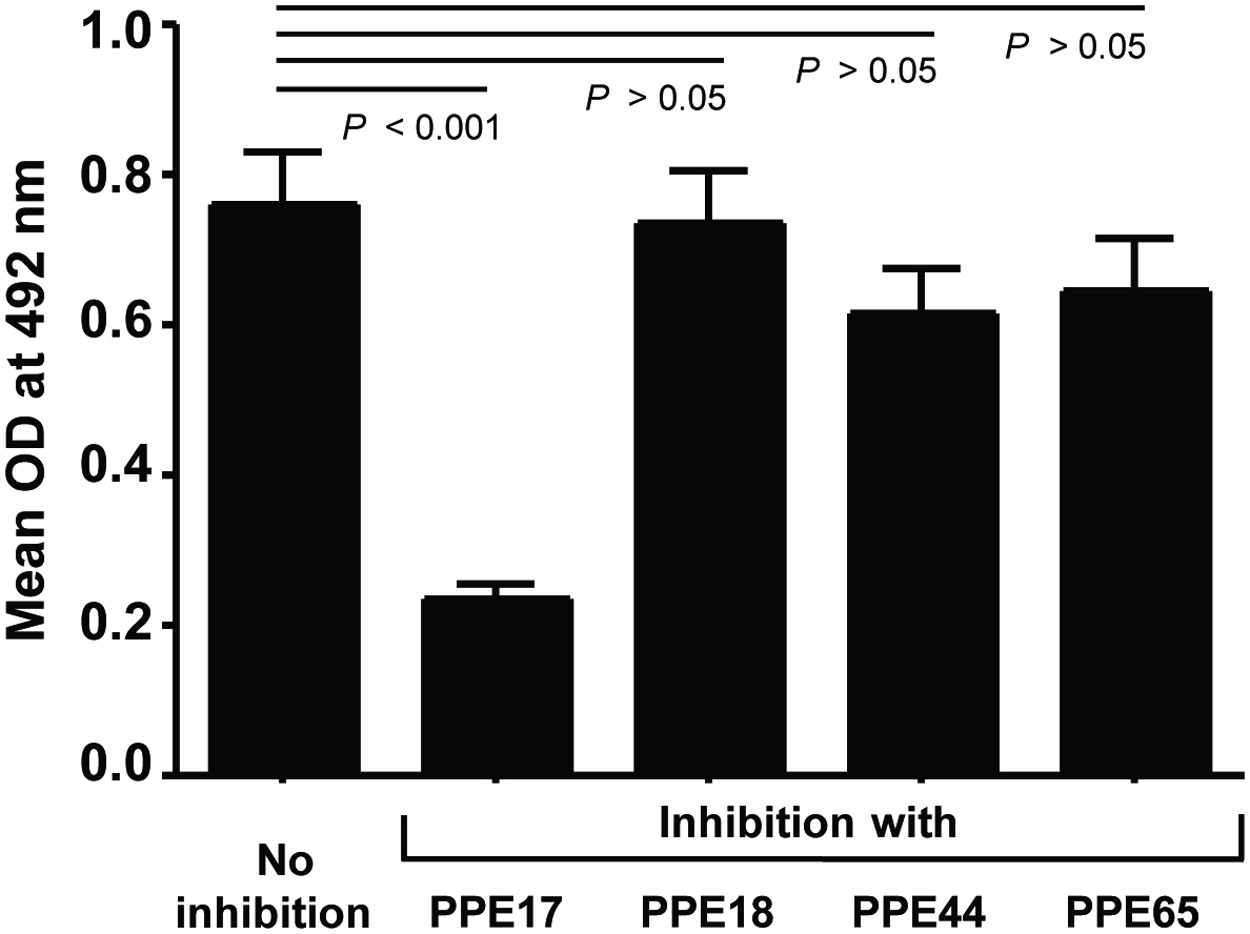
N-PPE17-specific antibodies in sera obtained from TB patients did not significantly cross-react with N-terminal domains of other PPE proteins. Sera samples (n = 50) pre-incubated with recombinantly purified full-length PPE17 or PPE18 or PPE44 or PPE65 protein were added to EIA plates coated with recombinantly purified N-PPE17. The plates were further incubated with anti-human IgG-HRP and absorbance was read at 492 nm using the chromogenic substrate OPD.

The N-terminal domain of PE/PPE family of proteins is known to be conserved [29, 31–33]. Since the antibody responses of active TB patients were induced predominantly against the N-terminal region of PPE17, it was of interest to examine whether N-PPE17 shared cross-reactivities with N-terminal domains of various other PPE proteins. Therefore, N-PPE17 antigen coated plates were incubated with TB patients sera either left untreated or pre-incubated with either full-length PPE17 (Rv1168c), full-length PPE18 (Rv1196), full-length PPE44 (Rv2770c) or full-length PPE65 (Rv3621c) proteins. These PPE proteins are known to be expressed during active TB infection and induce B-cell and T-cell immune responses [34–37]. The N-terminal domains of these PPE proteins share about 50% homology in the amino acid sequence with N-PPE17. Interestingly, pre-incubation of sera with PPE17 resulted in quenching of N-PPE17-specific antibody population up to 75% (*P* < 0.001) as expected, but the other three PPE proteins failed to significantly quench N-PPE17-specific antibodies (Fig 5; *P* > 0.05). This observation suggests that in TB patients, the N-terminal domain of PPE17 is immunodominant and N-PPE17-specific antibodies do not cross react with homologous N-terminal domains of other PPE proteins tested.

## Discussion

Since the discovery of *M*. *tuberculosis* PE/PPE proteins, several studies have been undertaken to identify suitable antigens that could be used to discriminate BCG-vaccinated healthy individuals from active TB patients [12,15,18,21,22]. We demonstrated earlier that the PPE17 protein could be used as a potent B-cell as well as T-cell marker to detect active TB disease [15]. In continuation of our efforts towards serological characterization of PPE17 in detail, we next studied the specific domain(s) of PPE17 that were responsible for induction of higher antibody responses in active TB patients. For this, the recombinant full-length PPE17 protein (PPE17) and N-terminal region of PPE17 (N-PPE17) proteins were used to screen the TB patient sera by EIA. Interestingly, we noted that the antibody responses generated in TB patients were mostly directed towards the N-PPE17, as in an inhibition EIA, pre-incubation of TB sera with the recombinantly purified N-PPE17 could significantly inhibit detection of full-length PPE17-specific antibody responses. It was observed that the patients with active TB disease had levels of N-PPE17-specific antibodies similar to that of full-length PPE17, indicating that the N-terminal domain of PPE17 was immunodominant. Further, PPE17-specific antibody levels in majority of the sera samples were neutralized by N-PPE17, suggesting that the N-terminal but not the C-terminal domain of PPE17 elicited stronger antibody responses in patients with active TB disease. This may be due to absence of immunodominant B-cell epitopes in the C-terminal domain of PPE17 protein. In our earlier studies, we observed that TB patients had higher anti-PPE17 antibody titers as compared to BCG-vaccinated healthy individuals [15]. In the present study, we demonstrate that antibody responses to PPE17 can be attributed to the PPE17 N-terminal domain in these patients. Both PPE17 and N-PPE17 were able to successfully discriminate sera of the active TB patients from the BCG-vaccinated healthy controls with sensitivities of 83.33% and 91.66% respectively. However, 16.66% (30/180) and 8.33% (15/180) patients showed OD below the cut-off values which could be due to differences in B and T-cell responses in patients which may be attributed to differences in individual genetic polymorphism [38–41].

Interestingly, when the immune response profiles of N-PPE17 in various clinical categories were studied, it was observed that the TB patients belonging to both pulmonary (smear positive and smear negative) and extrapulmonary clinical categories exhibited antibody responses to N-PPE17 equivalent to that of PPE17. These data reveal that all the three clinical categories of TB patients induce antibody responses predominantly against the N-PPE17 and N-PPE17 is probably associated with TB disease manifestation and can be used as a potent serodiagnostic marker to detect these categories of TB patients.

We have also compared the seroreactivity of TB patients against various concentrations of PPE17 and N-PPE17 proteins (ranging from 25 ng/well to 1μg/well) by EIA and the absorbance values were found to be well correlated with increased amount of PPE17 and N-PPE17 used. PPE17 as well as N-PPE17 proteins reacted strongly with serum antibodies from TB patients in EIA at 1 μg/well antigen concentration, however, at lower antigen concentrations (50 ng/well, 100 ng/well and 250 ng/well), N-PPE17 displayed higher absorbance than PPE17. This could be due to steric masking of N-terminal region in full-length PPE17. Thus, our study provides a hint that the N-terminal fragment of PPE17 could be used as a better serodiagnostic marker than the full-length PPE17 protein.

Since, the members of the PE/PPE family of proteins are characterized by highly conserved N-terminal domain, it is possible that the N-terminal epitopes of PPE proteins are responsible for antibody cross-reactivity in *M*. *tuberculosis*-infected patients. Therefore, we tested the cross-reactivity of N-PPE17 along with three other full-length PPE proteins such as PPE18 (Rv1196), PPE44 (Rv2770c) and PPE65 (Rv3621c). Sera samples of TB patients were pre-incubated with these proteins and then added to N-PPE17 coated EIA plates. Interestingly, we observed that none of the PPE proteins could significantly inhibit reactivity of sera samples to N-PPE17. Though there could exist a weak cross-reactivity to other N-terminal domains of PPE proteins, we hint at absence of a universal cross-reactive epitope in the N-terminal domains of PPE proteins used in this study. This indicates that though the N-terminal regions of PPE proteins share a conserved sequence, they may be structurally different. Earlier we demonstrated that N-terminal fragments of PPE17 and PPE18 proteins induce contrasting signaling cascades in macrophages due to their different conformations [30,35,42,43]. Thus, it is interesting to speculate that though the N-terminal region of about 180 amino acid residues of PPE proteins are conserved, the subtle variation in the sequence and length in the C-terminal region could change the three-dimensional epitopes of PPE proteins resulting in more unique immunoreactivity to N-PPE17. To test whether N-terminal domain of PPE17 has any unique region that is responsible for eliciting stronger antibody responses in TB patient sera, we used Imed antigenic prediction tool *(imed*.*meducm*.*es/Tools/antigenic*.*pi)* to predict the B-cell epitope present in the N-terminal domain of PPE17. When the predicted antigenic region of N-PPE17 was aligned with N-terminal regions of PPE18, PPE44 and PPE65 using EMBOSS water pair wise alignment tool, we found a stretch of amino acid sequence starting from 122 amino acid to 140 amino acid (IFGIHTPAIFALDALYAQY) is immunogenic and the percentage identity of this stretch with N-PPE18, N-PPE44 and N-PPE65 was only 38%, 36% and 42% respectively. Therefore, it can be concluded that the amino acid regions from 122 to 140 is almost 60% unique to N-PPE17 which might be responsible for the strong antibody response to PPE17 protein.

In summary, we demonstrate that the N-terminal domain of PPE17 protein is immunodominant and can discriminate BCG-vaccinated healthy controls from patients with active TB and can be used as a potent serodiagnostic marker. Though the present study involves samples from active TB patients only, it might be interesting to investigate application of the N-PPE17 protein antigen in discriminating individuals with latent TB infection from BCG-vaccinated healthy individuals.

## Supporting information

S1 Fig Amino acid sequence (1 to 346) of *Mycobacterium tuberculosis* PPE17 protein. Amino acid sequence starting from 1 to 173 (highlighted in bold) represents N-terminal region of PPE17. The predicted antigenic peptide (sequence starting from 122 to 140 amino acids) unique to N-terminal PPE17 is represented in the box.
(PDF)

S2 Fig Purification of recombinant PPE17 proteins. Recombinant PPE proteins from *Escherichia coli* were purified by affinity chromatography using TALON resins. Results shown is for Coomassie-blue stained sodium dodecyl sulfate gel showing the protein molecular weight marker (M) and elution fractions (1 to 7) for PPE17 (A), N-terminal fragment of PPE17 (B), PPE44 (C), PPE18 (D) and PPE65 (E). Molecular weight of the PPE proteins is highlighted with an arrow.
(PDF)

S3 Fig N-PPE17 inhibits detection of antibody response of TB patients to PPE17 in concentration-dependent manner. The EIA plate coated with either N-terminal fragment of PPE17 or full-length PPE17 antigen was incubated with 50 μl sera (*n* = 40) that were either left untreated or pre-incubated with 1μg (20 μg/ml) or 3 μg (60 μg/ml) of N-PPE17 protein. The plates were further incubated with anti-human IgG-HRP and absorbance was read at 492 nm using OPD.
(PDF)
