# Supplementary material for: The N-terminal domain of Mycobacterium tuberculosis PPE17 (Rv1168c) protein plays a dominant role in inducing antibody responses in active TB patients
Source: PLoS One. 2017 Jun 26;12(6):e0179965. doi: 10.1371/journal.pone.0179965 (PMC5484515; doi:10.1371/journal.pone.0179965)
Supplement: S1 Fig — Amino acid sequence starting from 1 to 173 (highlighted in bold) represents N-terminal region of PPE17. The predicted antigenic peptide (sequence starting from 122 to 140 amino acids) unique to N-terminal PPE17 is represented in the box. (PDF) [file pone.0179965.s001.pdf]

10 20 30 40 50

MDFTIFPPEF NSLNIQGSAR PFLVAANAWK NLSNELSYAA SRFESEINGL

60 70 80 90 100

ITSWRGPSST IMAAAVAPFR AWIVTTASLA ELVADHISVV AGAYEAAHAA

110 120 130 140 150

HVPLPVIETN RLTRLALATT NIFGIHTPAI FALDALYAQY WSQDGEAMNL

160 170 180 190 200

YATMAAAAAR LTPFSPPAPI ANPGALARLY ELIGSVSETV GSFAAPATKN

210 220 230 240 250

LPSKLWTLLT KGTYPLTAAR ISSIPVEYVL AFVEGSNMGQ MMGNLAMRSL

260 270 280 290 300

TPTLKGPLEL LPNAVRPAVS ATLGNADTIG GLSVPPSWVA DKSITPLAKA

310 320 330 340

VPTSAPGGPS GTSWAQLGLA SLAGGAVGAV AARTRSGVIL RSPAAG

S1 Fig. Amino acid sequence (1 to 346) of *Mycobacterium tuberculosis* PPE17 protein. Amino acid sequence starting from 1 to 173 (highlighted in bold) represents N-terminal region of PPE17. The predicted antigenic peptide (sequence starting from 122 to 140 amino acids) unique to N-terminal PPE17 is represented in the box.
