# Supplementary material for: The N-terminal domain of Mycobacterium tuberculosis PPE17 (Rv1168c) protein plays a dominant role in inducing antibody responses in active TB patients
Source: PLoS One. 2017 Jun 26;12(6):e0179965. doi: 10.1371/journal.pone.0179965 (PMC5484515; doi:10.1371/journal.pone.0179965)
Supplement: S2 Fig — Recombinant PPE proteins from Escherichia coli were purified by affinity chromatography using TALON resins. Results shown is for Coomassie-blue stained sodium dodecyl sulfate gel showing the protein molecular weight marker (M) and elution fractions (1 to 7) for PPE17 (A), N-terminal fragment of PPE17 (B), PPE44 (C), PPE18 (D) and PPE65 (E). Molecular weight of the PPE proteins is highlighted with an arrow. (PDF) [file pone.0179965.s002.pdf]

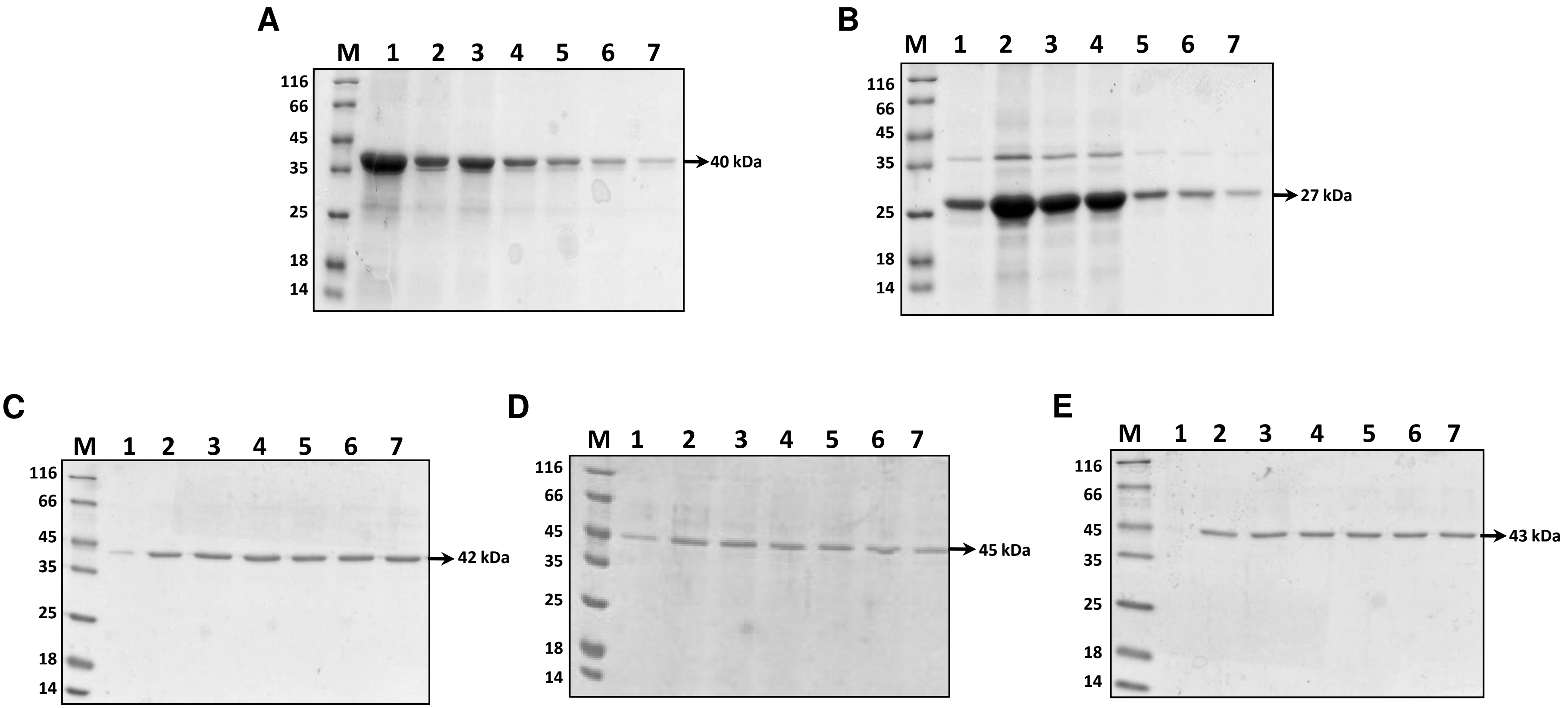

S2 Fig. Purification of recombinant PPE17 proteins. Recombinant PPE proteins from *Escherichia coli* were purified by affinity chromatography using TALON resins. Results shown is for Coomassie-blue stained sodium dodecyl sulfate gel showing the protein molecular weight marker (M) and elution fractions (1 to 7) for PPE17 (A), N-terminal fragment of PPE17 (B), PPE44 (C), PPE18 (D) and PPE65 (E). Molecular weight of the PPE proteins is highlighted with an arrow.
