# Supplementary material for: The N-terminal domain of Mycobacterium tuberculosis PPE17 (Rv1168c) protein plays a dominant role in inducing antibody responses in active TB patients
Source: PLoS One. 2017 Jun 26;12(6):e0179965. doi: 10.1371/journal.pone.0179965 (PMC5484515; doi:10.1371/journal.pone.0179965)
Supplement: S3 Fig — The EIA plate coated with either N-terminal fragment of PPE17 or full-length PPE17 antigen was incubated with 50 μl sera (n = 40) that were either left untreated or pre-incubated with 1μg (20 μg/ml) or 3 μg (60 μg/ml) of N-PPE17 protein. The plates were further incubated with anti-human IgG-HRP and absorbance was read at 492 nm using OPD. (PDF) [file pone.0179965.s003.pdf]

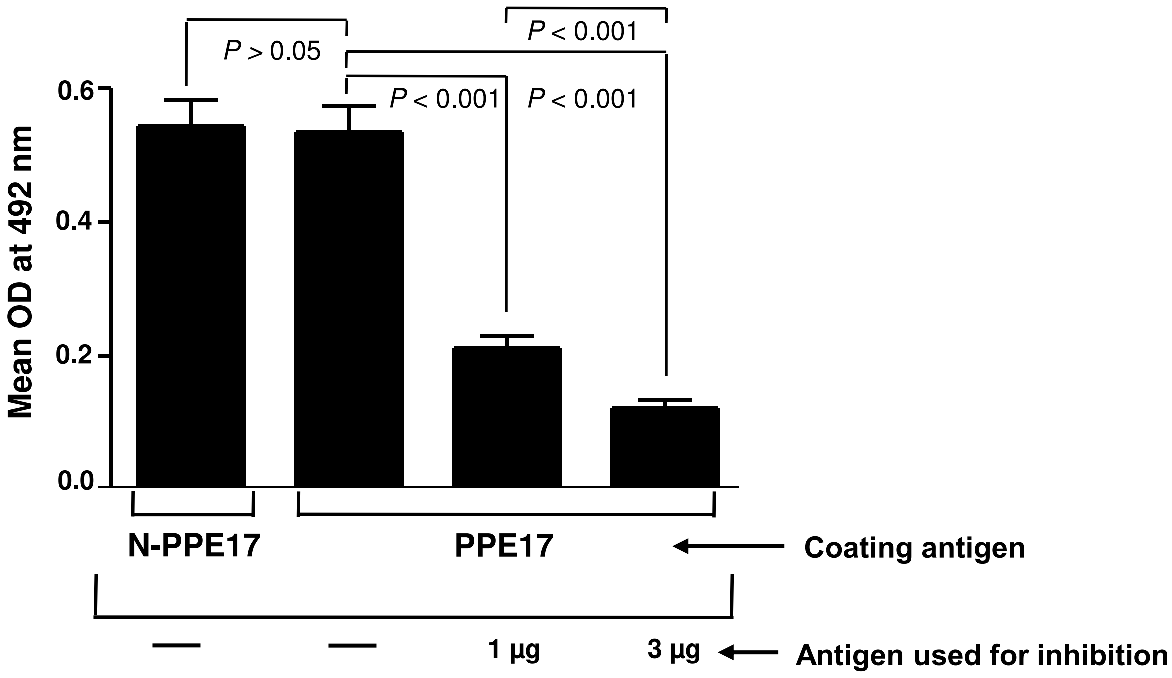

S3 Fig. N-PPE17 inhibits detection of antibody response of TB patients to PPE17 in concentration-dependent manner. The EIA plate coated with either N-terminal fragment of PPE17 or full-length PPE17 antigen was incubated with 50 µl sera (n = 40) that were either left untreated or pre-incubated with 1µg (20 µg/ml) or 3 µg (60 µg/ml) of N-PPE17 protein. The plates were further incubated with anti-human IgG-HRP and absorbance was read at 492 nm using OPD.
